# Endovascular comprehensive treatment of post-traumatic superior mesenteric arteriovenous fistula: case report and literature review

**DOI:** 10.3389/fcvm.2024.1414395

**Published:** 2024-06-26

**Authors:** Hongxin Wang, Kai Zheng, Qiangqiang Nie, Bo Yang, Xueqiang Fan, Peng Liu, Zhidong Ye

**Affiliations:** ^1^ Department of Interventional Therapy, The First Dongguan Affiliated Hospital of Guangdong Medical University, Dongguan, Guangdong, China; ^2^ China-Japan Friendship Hospital (Institute of Clinical Medical Sciences), Chinese Academy of Medical Sciences & Peking Union Medical College, Beijing, China; ^3^ Department of Cardiovascular Surgery, China-Japan Friendship Hospital, Beijing, China; ^4^ Department of Vascular Surgery, The First Affiliated Hospital of Zhengzhou University, Zhengzhou, Henan, China

**Keywords:** superior mesenteric arteriovenous fistula (SMAVF), endovascular treatment (EVT), coil embolization, covered stent, case report

## Abstract

**Background:**

Superior mesenteric arteriovenous fistula is a rare and difficult complication after abdominal trauma. Utilizing comprehensive endovascular treatment represents an effective approach to managing this condition.

**Case presentation:**

We report a case involving a 53-year-old female with a history of trauma who presented with complaints of abdominal pain, malaise, and melena. A computed tomographic scan revealed the presence of a superior mesenteric arteriovenous fistula. The fistula was occluded using four Interlock detachable coils, and a covered stent was positioned over the arteriovenous fistula in the superior mesenteric artery. Following endovascular treatment, the patient's abdominal pain and melena symptoms disappeared.

**Conclusion:**

Utilizing covered stents and Interlock detachable coils for endovascular treatment of a superior mesenteric arteriovenous fistula proves to be both feasible and highly effective.

## 1 Introduction

Superior mesenteric arteriovenous fistula (SMAVF) denotes an abnormal connection between the superior mesenteric artery and the superior mesenteric vein. This condition, though rare, is severe, with reported incidence rates at 0.09% and mortality rates ranging from 39% to 77%, primarily observed in young males (1). The formation of SMAVF can be congenital or occur secondary to trauma or abdominal surgery (2). Clinical manifestations of SMAVF encompass abdominal pain, bloating, diarrhea, gastrointestinal bleeding, and may even escalate to acute abdominal crisis (3). Diagnosis and treatment pose challenges due to the non-specific clinical manifestations of SMAVF.

Here, we present a case involving a female patient who developed a superior mesenteric arteriovenous fistula following abdominal trauma or gastrointestinal surgery. The fistula was effectively treated using four Interlock detachable coils and a covered stent.

## 2 Case report

### 2.1 Case description

A 53-year-old woman presented to our hospital with abdominal pain, primarily centered around the navel with no distinct localization, alleviated upon lying down. Over the past two weeks, symptoms have progressively exacerbated, accompanied by melena and anemia. Physical examination showed no remarkable findings. The patient had undergone enterectomy and anastomosis nine years ago following abdominal trauma.

### 2.2 Diagnostic assessment

**Figure 1:**
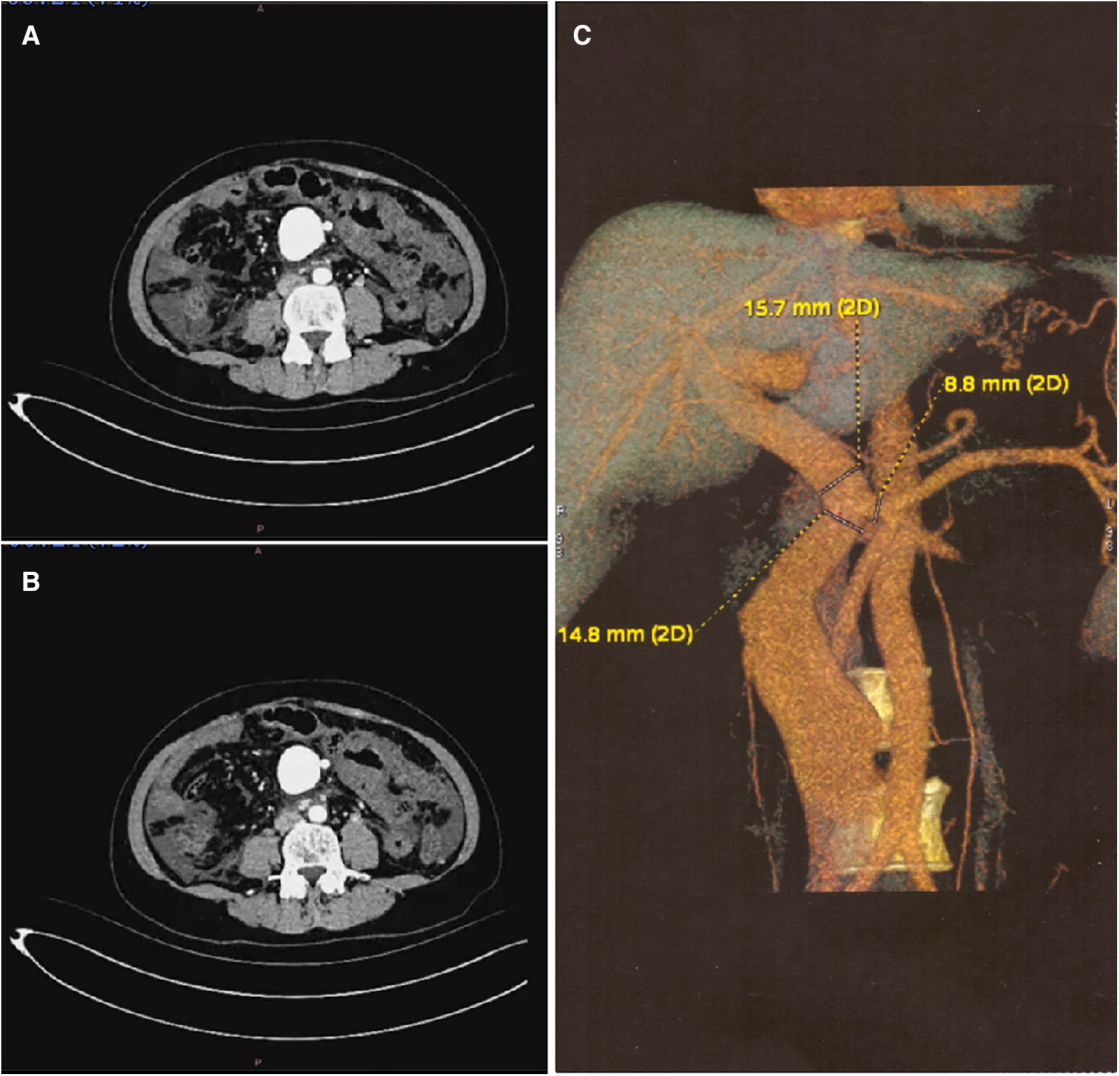
Preoperative Imaging Examination (**A,B**) CT imaging shows the superior mesenteric artery, fistula, and dilated superior mesenteric vein. (**C**) Three-Dimensional CT imaging shows the superior mesenteric artery, fistula, and dilated superior mesenteric vein.

The abdominal ultrasound revealed a dilated superior mesenteric vein with an inner diameter of approximately 22 mm and a flow rate of 49 cm/s. The portal vein appears slightly widened, with the main portal vein measuring approximately 15 mm in internal diameter. Computed tomography vascular reconstruction and angiography depicted abnormal arteriovenous flow and dilation of the superior mesenteric vein (Figure 1).

### 2.3 Treatment

**Figure 2:**
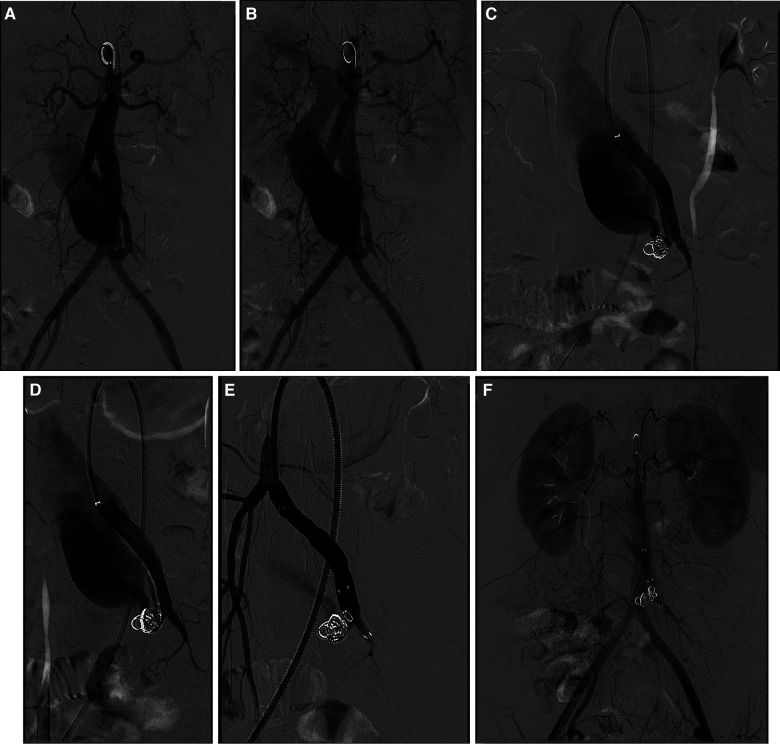
Digital subtraction angiography of endovascular procedure. (**A,B**) Baseline angiography. (**C**) Two Interlock coils were inserted for embolization. (**D**) Two more Interlock coils were inserted for embolization. (**E**) A covered stent was inserted. (**F**) Angiographic imaging at the end of the procedure.

The right femoral artery was accessed using the modified Seldinger technique, employing meticulous insertion of a 6 Fr arterial sheath (Cordis, Dublin, USA) and a 0.035-inch ultra-smooth guidewire (Terumo, Tokyo, Japan). The superior mesenteric artery was accessed using a guidewire and catheter system (Abbott, Chicago, USA). Subsequently, the guidewire was exchanged with a supercoil guidewire (Abbott, Chicago, USA), and a 6 Fr, 55 cm long sheath was introduced (Cook, Bloomington, USA). Digital subtraction angiography revealed abnormal communication between the distal ends of the superior mesenteric artery and vein, indicative of an arteriovenous fistula. The superior mesenteric vein exhibited abnormal dilation, measuring approximately 20 mm in diameter (Figures 2A,B). Subsequently, superselective catheterization was performed on the venous side of the fistula using a guidewire and catheter system (Boston Scientific, USA). Four Interlock detachable coils (Boston Scientific, USA) were sequentially deployed, resulting in partial relief of the fistula while maintaining patency (Figures 2C,D). Subsequently, a 7 Fr, 55 mm long sheath (Cook, Bloomington, USA) was repositioned, and the fistula was covered with a 5–60 mm Viabahn stent (Gore, Newark, USA) (Figure 2E). The distal and proximal ends of the stent were sequentially expanded using a 5–60 mm balloon (Cook, Bloomington, USA) (Figure 2E). Super-selective angiography revealed small branches of the superior mesenteric artery, with no evident shunt from the superior mesenteric artery to the superior mesenteric vein (Figure 2F).

### 2.4 Follow-up

Following endovascular treatment, the patient was advised to undergo dual antiplatelet therapy for 12 months, followed by lifelong single antiplatelet therapy. Subsequently, the patient experienced significant improvement in symptoms, cessation of gastrointestinal bleeding, and notable reduction in abdominal pain. At the three-month follow-up, abdominal ultrasound revealed patent superior mesenteric and main portal veins, with respective lumen diameters of 6.3 mm and 12 mm. Satisfactory clinical outcomes were achieved, and no complications were noted during follow-up.

## 3 Discussion

SMAVF is exceptionally rare in clinical practice. The inaugural case of a superior mesenteric arteriovenous fistula was documented by Movitz and Finnel in the 1960s (4). To date, fewer than 50 cases have been reported globally (5, 6). SMAVF typically arises subsequent to trauma or abdominal surgeries, such as stabbing incidents, ileectomy, or right hemicolectomy procedures. Diagnosis is frequently delayed as SMAVF often remains asymptomatic for several years. Patients with SMAVF commonly present with delayed symptoms, which may manifest up to fourteen years following bowel resection (5). The most commonly reported clinical symptom is mesenteric angina, characterized by abdominal pain and diarrhea occurring after meals. Less frequently, elevated portal venous pressure can lead to hepatic congestion, venous aneurysms, ascites, and esophageal varices (7). Due to its nonspecific presentation, including asymptomatic cases in the initial stages, SMAVF is prone to misdiagnosis.

Imaging plays a pivotal role in diagnosing, characterizing, and guiding management of SMAVF. Various methods are available for visualizing an SMAVF. Percutaneous abdominal angiography stands as the gold standard for delineating the location and extent of mesenteric vascular involvement (5). Given its invasiveness, percutaneous abdominal angiography is typically reserved for treatment purposes. Instead, abdominal CT angiography, three-dimensional angiography, and MRI serve as the most reliable modalities for diagnosing the disease (8).

Superior mesenteric arteriovenous fistulas can be classified into U type and H type. U type typically results from iatrogenic injury, wherein the superior mesenteric artery or its branches directly connect with the vein. H type, more prevalent in traumatic SMAVF, involves the local formation of a false aneurysm in the superior mesenteric artery or its branches, which then connects with a vein (9). In this case, surgical confirmation revealed collateral blood flow reflux at the patient's distal end, forming a phlebotoma with a diameter of approximately 2 cm. The arteriovenous fistula between the superior mesenteric artery and vein was classified as H type.

SMAVFs can be managed through either surgery or endovascular treatment. Open repair has traditionally been considered the preferred option for high-flow intra-abdominal AVFs (10). However, with advancements in endovascular technology and embolic materials, embolization has emerged as the preferred treatment for superior mesenteric arteriovenous fistulas (11). Since the pioneering report by Uflacker and Saadi (12), there have been 50 documented cases of endovascular treatment for iatrogenic superior mesenteric AVFs (13). The objective of endovascular treatment is to close the fistula while preserving the branches of the SMA. When selecting an appropriate embolization method, consideration should be given to the type of fistula: U-type fistulas lack distal intestinal supply, whereas H-type fistulas have a distal intestinal supply (14, 15). In our case, the initial embolization with two coils did not yield satisfactory results, prompting the addition of two more coils. It is essential for surgeons to accurately measure the diameter of the feeding artery and meticulously deploy the coils at the target artery to prevent coil migration and avoid complications such as distal mesenteric arterial occlusion or portomesenteric venous thrombosis.

Early attempts at endovascular intervention for SMAVF primarily utilized coil embolization of the aberrant branches. However, in certain cases, the use of covered stents is recommended due to the significant risks associated with coil embolization, including coil migration, distal enteric arterial occlusion, or porto-mesenteric venous thrombosis (5, 16). In this patient's case, the initial embolization with four Interlock detachable coils did not immediately narrow the dilated superior mesenteric vein. Consequently, a covered stent was deployed to occlude the fistula. When using covered stents, it is crucial to ensure optimal stent expansion and adequate apposition to the vessel wall to minimize the risk of intra-stent thrombosis. Placement of a balloon-expandable stent across a sharp bend should be approached with caution to avoid abnormal vessel straightening, vessel injury, or stent deformation (17, 18). In this case, there was no angiographic evidence of the fistula following placement of the covered stent, and subsequent resolution of symptoms was observed.

Endovascular stent placement is associated with an elevated risk of thrombosis (19, 20). Empirically, dual antiplatelet therapy is recommended postoperatively, followed by lifelong treatment with aspirin. However, the optimal duration of dual antiplatelet therapy remains uncertain. For SMAVF, there is insufficient evidence to advocate for routine anticoagulant administration (1, 21). In this case, the patient was advised to undergo dual antiplatelet therapy for 12 months, followed by a lifelong single antiplatelet regimen.

## 4 Conclusion

In conclusion, coils embolization and covered stents represent minimally invasive and safe treatment options for SMAVF, with significant effectiveness. Open surgery is typically reserved as a secondary option following the failure of vascular interventional procedures, mainly due to its associated trauma. However, further observation and evaluation are warranted to assess the long-term outcomes and effectiveness of this endovascular approach in treating SMA and SMV.

## Data Availability

The original contributions presented in the study are included in the article/**Supplementary Material**, further inquiries can be directed to the corresponding authors.
