# Supplementary figures and images for: Endovascular comprehensive treatment of post-traumatic superior mesenteric arteriovenous fistula: case report and literature review
Source: Front Cardiovasc Med. 2024 Jun 26;11:1414395. doi: 10.3389/fcvm.2024.1414395 (PMC11233816; doi:10.3389/fcvm.2024.1414395)

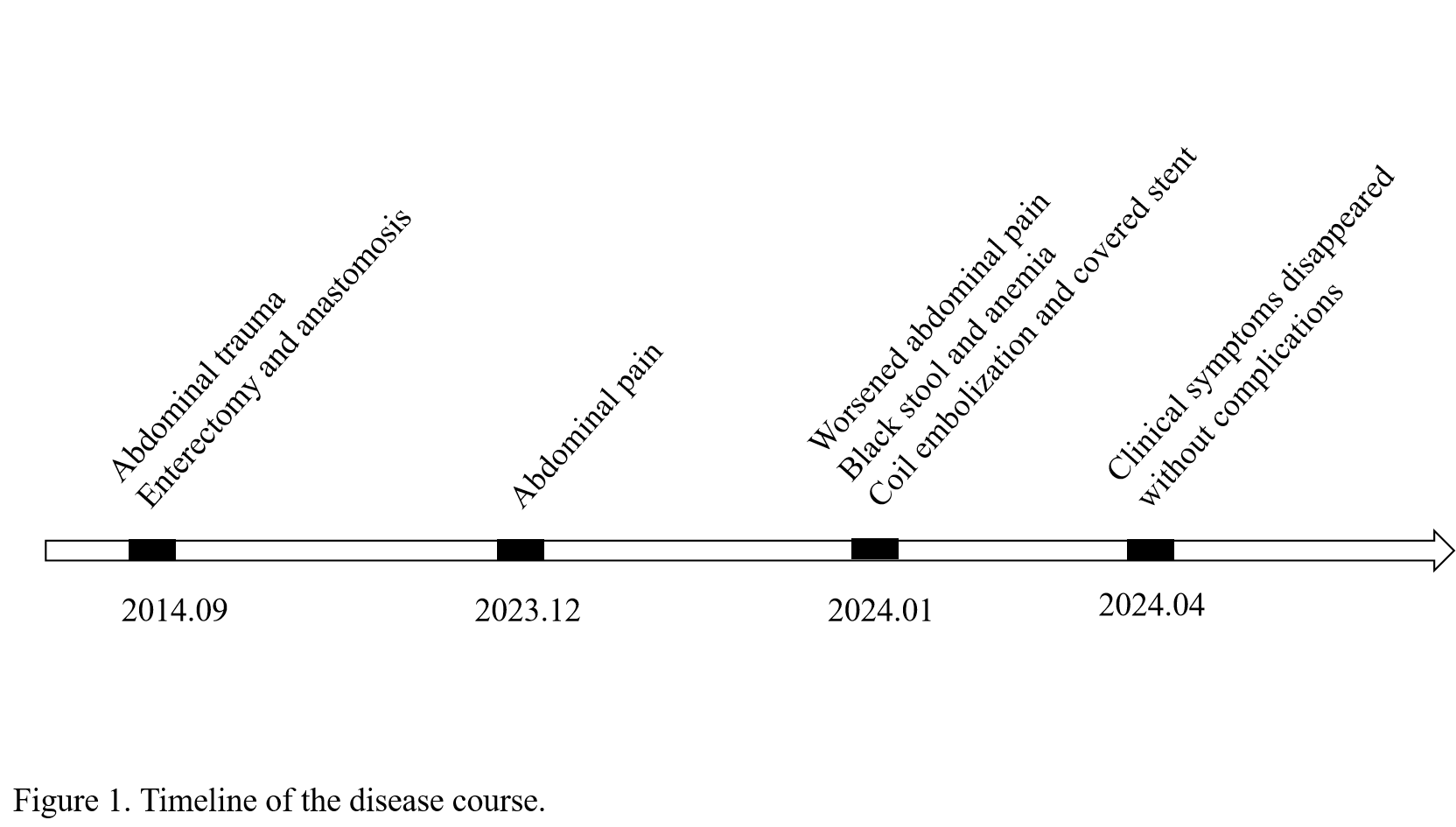

Supplement: Supplementary file 1 [file Image1.png]
